# Transfer-Learning Deep Radiomics and Hand-Crafted Radiomics for Classifying Lymph Nodes from Contrast-Enhanced Computed Tomography in Lung Cancer

**DOI:** 10.3390/cancers15102850

**Published:** 2023-05-21

**Authors:** Fabian Christopher Laqua, Piotr Woznicki, Thorsten A. Bley, Mirjam Schöneck, Miriam Rinneburger, Mathilda Weisthoff, Matthias Schmidt, Thorsten Persigehl, Andra-Iza Iuga, Bettina Baeßler

**Affiliations:** 1 Department of Diagnostic and Interventional Radiology, University Hospital Würzburg, University of Würzburg, 97080 Würzburg, Germany; 2 Institute of Diagnostic and Interventional Radiology, Medical Faculty and University Hospital Cologne, University of Cologne, 50937 Cologne, Germany; 3 Department of Nuclear Medicine, Medical Faculty and University Hospital Cologne, University of Cologne, 50937 Cologne, Germany

**Keywords:** computed tomography, computational neural networks, lymphatic metastasis, carcinoma, non-small-cell lung, small-cell lung

## Abstract

**Simple Summary:**

Positron emission tomography is currently considered the non-invasive reference standard for determining whether lung cancer also affects thoracic lymph nodes (staging). However, not all patients can undergo this diagnostic procedure due to high costs, limited availability, and additional radiation exposure. This study aimed to predict the positron emission tomography result from traditional contrast-enhanced computed tomography and test new feature extraction strategies. As input, we compared traditional (hand-crafted) imaging biomarkers (radiomics) with novel features derived from pre-trained neural networks. This hybrid approach yielded better performance than using both feature sources alone. In conclusion, both traditional radiomics features and transfer-learning deep radiomics features provide relevant and complementary information for non-invasive lymph nodal staging in lung cancer.

**Abstract:**

Objectives: Positron emission tomography (PET) is currently considered the non-invasive reference standard for lymph node (N-)staging in lung cancer. However, not all patients can undergo this diagnostic procedure due to high costs, limited availability, and additional radiation exposure. The purpose of this study was to predict the PET result from traditional contrast-enhanced computed tomography (CT) and to test different feature extraction strategies. Methods: In this study, 100 lung cancer patients underwent a contrast-enhanced ^18^F-fluorodeoxyglucose (FDG) PET/CT scan between August 2012 and December 2019. We trained machine learning models to predict FDG uptake in the subsequent PET scan. Model inputs were composed of (i) traditional “hand-crafted” radiomics features from the segmented lymph nodes, (ii) deep features derived from a pretrained EfficientNet-CNN, and (iii) a hybrid approach combining (i) and (ii). Results: In total, 2734 lymph nodes [555 (20.3%) PET-positive] from 100 patients [49% female; mean age 65, SD: 14] with lung cancer (60% adenocarcinoma, 21% plate epithelial carcinoma, 8% small-cell lung cancer) were included in this study. The area under the receiver operating characteristic curve (AUC) ranged from 0.79 to 0.87, and the scaled Brier score (SBS) ranged from 16 to 36%. The random forest model (iii) yielded the best results [AUC 0.871 (0.865–0.878), SBS 35.8 (34.2–37.2)] and had significantly higher model performance than both approaches alone (AUC: *p* < 0.001, z = 8.8 and z = 22.4; SBS: *p* < 0.001, z = 11.4 and z = 26.6, against (i) and (ii), respectively). Conclusion: Both traditional radiomics features and transfer-learning deep radiomics features provide relevant and complementary information for non-invasive N-staging in lung cancer.

## 1. Introduction

Lung cancer is the most frequent cause of death in developed countries. The status of the disease stage (including the presence or absence of loco-regional lymph node metastases) at the time of diagnosis is highly relevant for treatment choice and the expected outcome [1].

The non-invasive reference standard for nodal (N-) staging in lung cancer is ^18^F-fluorodeoxyglucose positron emission tomography/computed tomography (^18^FDG-PET/CT) [1,2,3]. However, high costs, limited availability, and additional radiation exposure prevent its widespread application among lung cancer patients worldwide. A non-invasive alternative using only the information from routinely acquired contrast-enhanced CT (CECT) would thus be highly needed.

Radiomics and machine learning have been successfully applied to CECT to predict the severity of lesions in various organs [4]. In lung cancer, several studies showed the potential of this approach for detecting and classifying pulmonary nodules and masses [4,5,6,7]. However, until now, the demanding manual segmentation, small sample sizes from single centers, and limited availability of standardized outcomes prevented general applicability and translation into clinical practice [4,5,6,8].

Moreover, there are also technical challenges related to the application of radiomics. “Traditional” hand-crafted radiomics features are derived from statistical estimators that humans can interpret and are not optimized for machine consumption. They are highly correlated and often contain redundant information [9,10,11]. Despite efforts to standardize radiomics (e.g., IBSI [12]), the lack of robustness limits the generalizability of the radiomics approach [9,10].

In contrast to hand-crafted radiomics features, most state-of-the-art methods for image classification and segmentation in computer vision are based on convolutional neural networks (CNN). Here, the outputs of the first layers of the neural network are a latent representation of the respective image features but are intrinsically optimized for machine consumption in subsequent layers [13,14].

“Deep” features can be extracted from the output of the first layers of a pre-trained CNN and treated similarly to hand-crafted radiomics in subsequent machine learning models [13,14]. First studies tested this approach in classifying different disease entities with promising results and showed good robustness of deep features [13,14,15]. However, the complementary value of combining it with traditional radiomics features and its application to the classification of lymph nodes have not yet been investigated.

This study aimed to build and evaluate a classification model that predicts lymph node dignity in lung cancer from radiomic tissue characteristics derived from CECT and compare different feature extraction strategies using FDG-PET/CT as the reference standard. The primary goal of our study was to use this classification task as an example use case to demonstrate the impact of different feature extraction strategies on an exemplary machine learning classification task.

## 2. Methods

### 2.1. Study Sample and Design

**Figure 1:**
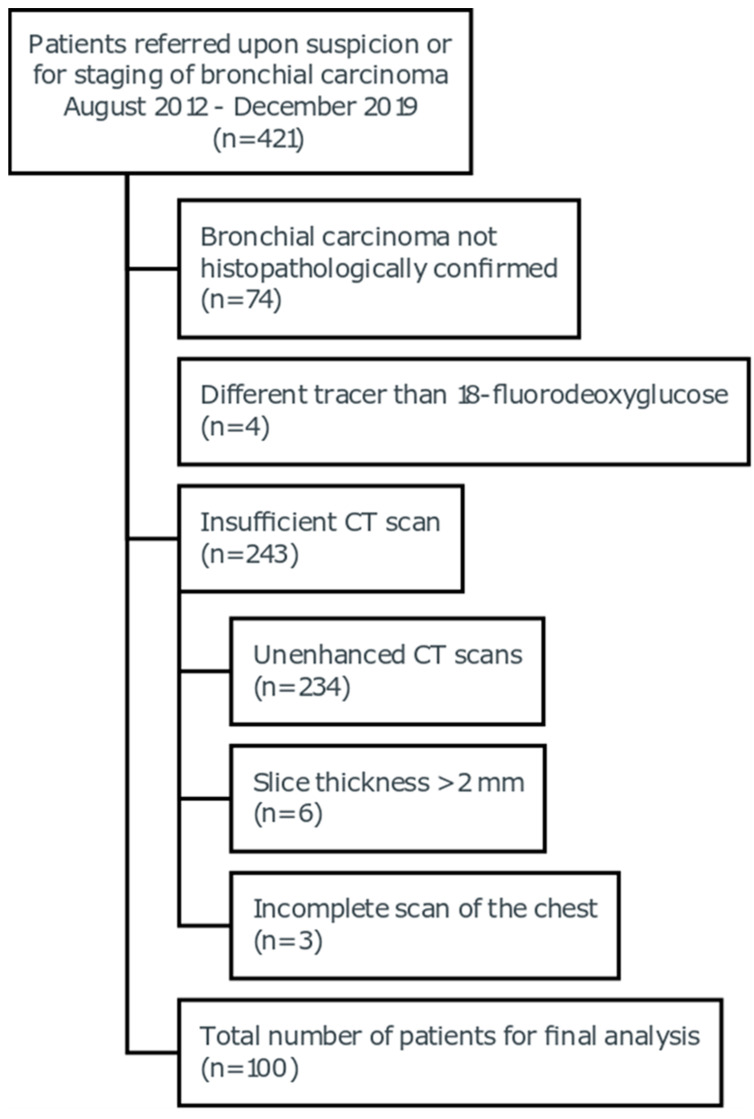
CONSORT flowchart of the analyzed study population. CT = computed tomography.

In this study, 100 patients with histopathologically confirmed lung cancer who were referred upon suspicion or for the staging of bronchial carcinoma from August 2012 to December 2019 were included. A flowchart stating the reason for the exclusion of patients is given in Figure 1. All subjects received an FDG-PET/CT scan simultaneously with a contrast-enhanced CT scan. All examinations were performed on a 128-slice PET/CT system (Siemens Biograph mCT Flow 128 Edge, Siemens Healthineers, Erlangen, Germany). Patients were scanned supine in craniocaudal direction during inspirational breath-hold after intravenous injection of 120 mL of contrast medium (Accupaque 350, GE Healthcare, Boston, MA, USA) with an injection rate of 2.5 mL/s and a delay of 60 s. The following scan parameters were used: collimation 128 × 0.6 mm, rotation time 0.5 s, and pitch 0.6. All axial images were reconstructed with a slice thickness of 2 mm.

Ethical approval was waived due to the study’s retrospective design based on preexisting images (Ethics Committee reference number 19-1379/16.08.2019).

### 2.2. Segmentation and Image Preprocessing

A radiologist (AII; 6 years of experience in thoracic imaging) manually segmented all thoracic lymph nodes with a short-axis diameter of at least 5 mm from the included contrast-enhanced CT scans (n = 100). The 3D volume of all axillary, mediastinal, and hilar lymph nodes was segmented using the semi-automatic 3-dimensional Multi-Modal Tumor Tracking tool of a commercially available software platform (IntelliSpace Discovery, Version 3.0.5, Philips Healthcare Amsterdam, The Netherlands).

Additionally, lymph nodes with FDG uptake above liver niveau in the FDG-PET/CT scans were separately labeled (PET-positive lymph nodes).

Unclear findings were discussed with a radiologist with 16 years of experience in oncological imaging, including FDG-PET/CT imaging (TP).

These 3-dimensional segmentations served as the volume of interest (VOI) for the radiomic feature extraction. Images were resampled to isometric voxels with 1 mm spacing. No scaling was applied to the image intensities measured in Hounsfield units. Feature maps, used to better interpret the results, were calculated using voxel-based extraction with pyradiomics for hand-crafted features and direct values from the third layer of the CNN for deep features. For voxel-based feature extraction, the features are calculated for a specified neighborhood around each individual voxel within the region of interest (ROI), resulting in feature maps representing the spatial distribution of the features. The configuration file for the pyradiomics package will be part of the publicly available source code.

### 2.3. Machine Learning

An L1- and L2-penalized (‘elastic’) logistic regression model was trained to predict whether a lymph node showed FDG uptake on the subsequent PET/CT scan (i.e., was PET-positive). As a sensitivity analysis, we applied a random forest, which is in contrast to the ‘elastic’ logistic regression and is also capable of learning non-linear and interactive relations between the features and the outcome. In addition, we use a gradient-boosted tree model (XGBoost) that is documented in the Online Supplements. We applied the principal component analysis as an unsupervised dimension reduction and decorrelation method. A linear transformation transforms the input feature vector into a new space of orthogonal (or decorrelated) eigenvectors. In the first step, the dimensionality is still maintained. However, by choosing only the first—say, L—eigenvectors by a given criterion, dimensionality can be reduced. We did not set a fixed number of kept eigenvectors but chose L so that the reconstruction using the new set of eigenvectors explains 95% of the variance in the original data. We tested model hyperparameters in parallel using a random search approach.

We compared different feature extraction methods (cf. Table 1).

All experiments were carried out on 40 nodes of a high-performance computation cluster in parallel using SLURM. Per node, 70 CPU cores and 96 GB of RAM were allocated. We implemented the experiments in Python 3.7.9 with the additional packages scikit-learn 1.0.2, pyradiomics 3.0.1, AutoRadiomics 1.0, and scipy 1.7.3.

The models and the respective hyperparameter configurations were trained and evaluated in a 10-times-repeated, 20-fold cross-validation. We applied the splits on the patient level (i.e., either all patient lymph nodes or none were in the respective training set) and a bootstrap correction on the pooled out-of-bag predictions to account for the optimistic bias due to the testing of multiple hyperparameters [18].

### 2.4. Statistical Analysis and Performance Evaluation

Discriminatory performance was visually assessed by the receiver operating characteristics (ROC) and quantitatively assessed by the corresponding area under the receiver operating characteristics (AUC).

The AUC describes the discrimination performance, which measures whether a PET-positive lymph node has a higher predicted probability than a PET-negative lymph node. A value of 1.0 means perfect discrimination. If the model had no discriminative ability (i.e., toss of a coin) in the investigated population, this would result in an AUC of 0.5. AUC values below 0.5 occur if the model predicts an informative but wrong ordering.

We calculated the sensitivity and specificity of the models for a cutoff chosen to maximize the Youden index (sensitivity + specificity).

In addition, we evaluated model calibration and absolute inaccuracy using lowess-smoothed calibration plots, the mean square error (Brier score), and a scaled *R*^2^-like variant $R^{2}=1-\frac{BS}{BS_{ref}}$. Here, $BS_{ref}$ is the Brier score for a naïve model (i.e., a model that always predicts the average outcome frequency in the training sample).

The scaled Brier score (SBS) gives the fraction by which the mean square error is reduced compared to an uninformative model (i.e., predicting the same probability of being PET-positive for every lymph node without considering any specific information). A perfect SBS equals 100%. An SBS of 0% means that the model provides no information benefit. SBS below 0 means the prediction error is even higher (e.g., because the model is miscalibrated) than a naïve calibrated model. To avoid artificially introduced miscalibration, we did not add weighting to account for imbalanced binary classes.

A two-sided bootstrapped z-test was applied to differences in BS and AUC. We used quantile-quantile plots and kernel density histograms to check the normality assumption. *p* < 0.05 was considered the threshold for statistical significance. Due to the nature of this exploratory study, we did not correct for multiple testing [19].

We used STATA 15.1 (StataCorp, College Station, TX, USA) to carry out statistics on the study population.

## 3. Results

### 3.1. Study Population

In total, we included 2734 lymph nodes (555, 20.3% PET-positive) from 100 patients [49% female, median age 65 years (SD 10)] with lung cancer in this study. Baseline characteristics of the study population are given in Table 2.

### 3.2. Hand-Crafted, Deep, and Hybrid Features

**Figure 2:**
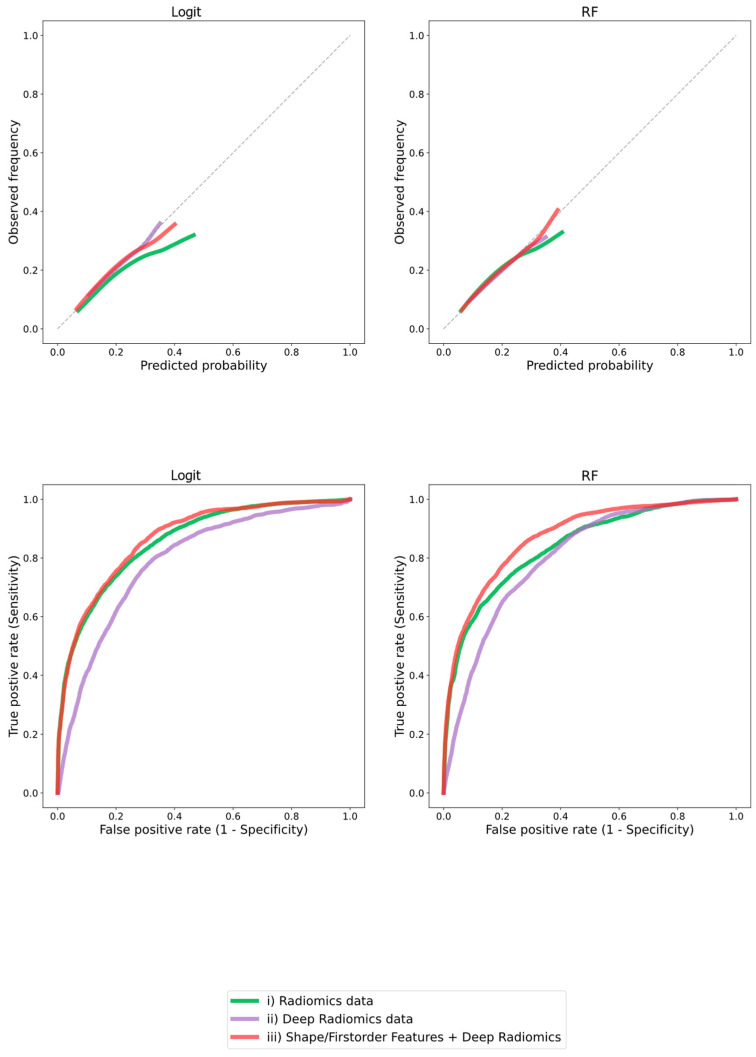
Calibration and discrimination for classification of PET positivity for different radiomic approaches. Results of penalized logistic regression (logit) and random forest are shown. In the lowess-smoothed calibration plot (first row), the observed outcome frequency is plotted against the predicted outcome probability. The closer the curve is to the diagonal, the better the calibration. The receiver operating characteristic (second row) plots the true positive rate against the false positive rate by varying thresholds (not shown). Discrimination is best for the curve that is closest to the left upper corner. Legend: PCA = principal component analysis; RF = random forest.

Compared to “traditional” hand-crafted radiomics features, the model discrimination and overall prediction error of the logit models trained on deep CNN features alone were slightly worse (*p* < 0.001, z = 8.8 and *p* < 0.001, z = 14.1 for AUC and BS, respectively) but still informative (Table 3, Figure 2).

Combining hand-crafted first-order and shape features with deep features (iii) resulted in significantly improved overall prediction error (BS; *p* < 0.001, z = 11.4 and z = 26.6 against (i) and deep (ii) alone, respectively; Figure 2, Table 3) compared to either approach alone.

The models’ discrimination (AUC) was only improved in comparison to (ii) deep features alone (*p* < 0.001, z = 25.2) but not to the (i) hand-crafted radiomics features model (*p* = 0.12, z = 1.6, second row of Figure 2, Table 3). A difference in discriminatory capability was visible in the ROC curve (second row of Figure 2), favoring model (iii) over the other two models. In addition, the sensitivity and specificity differ across models due to differing thresholds selected by the Youden-index criterion.

The calibration of the models (first row of Figure 2) was visually better (closer to the diagonal) for the models (ii) and (iii) compared to (i).

**Figure 3:**
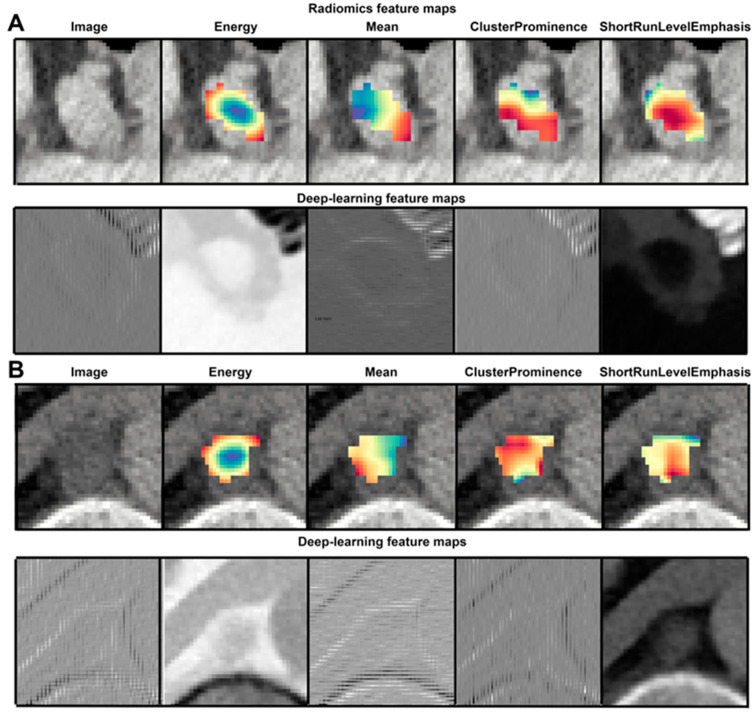
Visualization of hand-crafted radiomic features and deep features. (**A**) is an example of a PET-positive lymph node. (**B**) is an example of a PET-negative lymph node. Selected hand-crafted and random selections of feature map outputs of the third layer of the deep CNN are presented, respectively. Sensitivity analysis: random forest.

Figure 3 illustrates the hand-crafted and deep radiomics features for a PET-positive and a PET-negative lymph node, respectively.

The random forest model, capable of learning non-linear and interactive relationships, resulted in lower or equal Brier scores for all models (Figure 2, Table 3). For example, for model (iii), the BS was significantly lower (*p* = 0.005, z = 2.8) for the RF model compared to the logit model. In contrast, there was no significant difference (*p* = 0.09, b = 1.7) between the AUC of the RF and the logit variant of model (iii), while the AUC of model (i) was significantly (*p* = 0.009, b = −2.6) higher for the logit variant of model (i). An additional experiment using XGBoost with similar results is documented in the Online Supplements (Table S1, Figure S1).

Compared to both approaches alone, combining hand-crafted first-order and shape features with deep features resulted in significantly improved model discrimination (AUC; *p* < 0.001, z = 8.8 and *p* < 0.001, z = 22.4 against the traditional hand-crafted and deep features alone, respectively) and overall prediction error (BS; *p* < 0.001, z = 11.4 and z = 26.6 against the traditional hand-crafted and deep features alone, respectively). Visually, the ROC curve and the calibration curve (right side of Figure 2) were also best for model (iii).

Among all tested classifiers, the best-performing model was a random forest classification for the (iii) hybrid features.

## 4. Discussion

To our knowledge, this is the first study that analyzes advanced feature extraction techniques—namely deep radiomics features extracted from a pre-trained 2D CNN—in the prediction of dignity in thoracic lymph nodes in lung cancer patients. Overall, discrimination of PET-positive and PET-negative lymph nodes from radiomic tissue characteristics was excellent, with AUCs ranging from 0.78 to 0.88 and a reduction in the prediction error of up to 36%. Interestingly, combining “traditional” hand-crafted first-order and shape features with deep features derived from a pre-trained CNN resulted in significantly lower prediction errors than both approaches alone.

This work shows that deep features derived from a pre-trained CNN could be used to discriminate PET-positive from PET-negative lymph nodes. However, the preprocessing (scaling and resampling) precludes the use of information on the absolute attenuation of tissues and the size of lymph nodes. The latter is what a human would use for the assessment of dignity and is used in many diagnostic criteria [20,21].

Furthermore, the lack of absolute information masks the distinct attenuation characteristic. For example, identifying fatty tissue with characteristic negative Hounsfield units corresponding to the so-called “positive hilus sign” indicating a benign lymph node [22] may be impossible using the deep features. Hence, it is not surprising that adding the first-order features and the shape features containing information about the absolute attenuation and the size significantly improves model performance.

Similar studies have tried to predict the nodal status of lung cancer and other disease entities [23,24,25]. The authors of [23] applied a LASSO [26] model to directly estimate the N-stage on a per-patient basis using clinical information and features derived from PET/CT. They evaluated the model discrimination, not the calibration, in a single train/test split and a small external validation cohort. In contrast, we used information from CECT only to predict the lymph node dignity for each thoracic lymph node. Furthermore, our evaluation framework using bootstrap-bias-corrected 10-times-repeated 20-fold cross-validation accounts for the variance introduced by an arbitrary single train/test split in small sample sizes [18,27] and measures both calibration and discrimination [27]. Other authors applied radiomics to primary pulmonary neoplasms and metastases in CECT and PET/CT [28,29,30,31,32,33]. Predicted outcomes were composed of the severity of pulmonary lesions, epidermal growth factor receptor status, or survival outcomes [28,29,30,31,32,33]. In contrast, we focus on the per-node dignity of thoracic lymph nodes, where rather little research has been conducted [23,24]. However, the potential synergies of applying radiomics and machine learning to the primary tumor and the thoracic lymph nodes remain subject to future research. In clinical practice, a suspicious lymph node in lung cancer patients requires a specific workup. Guidelines recommend PET-CT if available [1]. If treatment-relevant uncertainty remains afterward, a biopsy (transbronchial, transcutaneous, or thoracoscopic) is recommended [1]. Since there is no generally accepted established threshold, this decision is usually found in the consensus of an interdisciplinary cancer board. Here, experts assess the probability of a lymph node being malignant based on image features and patient characteristics. Similarly, the output of the machine learning models is not a binary decision. Instead, a probability of PET positivity for each individual lymph node is provided and may be considered when PET or CT is unavailable and aids further work-up. However, from our point of view, metabolic FDG-PET/CT seems to be the most specific imaging technique in non-invasive lung cancer N-staging, but radiomics could improve conventional DECT interpretation in the case of missing FDG-PET/CT.

In the sensitivity analysis concerning the assumptions on linearity and absence of interactive effects in the elastic logistic regression, the more flexible random forest had a significantly lower overall prediction error (BS) compared to the penalized logit model when using both hand-crafted and deep features as a model input (iii). In contrast, there was only a non-significant difference in AUCs between the RF and logit models. For hand-crafted features (i), the AUC for RF was significantly lower than for the logit model. BS measures discrimination and calibration, while AUC measures only the former [34,35,36]. Since discrimination and calibration are both important for clinical decisions, we chose BS as the primary metric. Optimizing hyperparameters for BS leads to the selection of hyperparameter configurations that favor calibration for the RF and discrimination for the logit models [35]. This can also be seen in the calibration plots (first row of Figure 2), indicated by more deviation from the diagonal for the logit models. Both the results for the elastic net logistic regression and random forest support the hypothesis that the hybrid approach (iii) is superior to hand-crafted (i) and deep features (ii) alone.

The “identification” of single imaging biomarkers by feature selection is common practice in biomedical “omics” analysis [37]. However, as opposed to metabolomics, genetic or molecular biomarkers, hand-crafted features, and CNN-based features are not potential treatment targets. In addition, there is no additional cost caused by a higher number of features. Either manual, AI-assisted, or fully automatic segmentation of the to-be-classified target structure was conducted, and hence all the features would be available or not. Therefore, reducing the potential feature space may only be relevant for the subsequent training of machine learning models for the specified task. The potential benefit may only be assessed in terms of the models’ performance. Preferably, this process should be conducted as part of the model training and hyperparameter optimization. In our study, we used an embedded supervised approach (elastic net [38]) and combined it with an unsupervised feature reduction method (PCA).

CNNs are usually considered “black boxes” [39]. However, modern model inspection techniques and the demonstrated way to visualize the deep features extracted from CNNs (Figure 3) simplify human interpretation [14]. Moreover, reproducibility is considered a prerequisite for generalizability. Other studies showed superior reproducibility of CNN-derived deep features compared to hand-crafted radiomic features [13].

We foster the reproducibility of the experiments conducted using only publicly available open-source packages. The experiments’ Python code will be made publicly available and can be retrieved from github.com/laqua-stack/BC_CNN_Radiomics. The hand-crafted features extracted using the pyradiomics package comply with the feature definitions of the Image Biomarker Standardization Initiative [12]. Furthermore, applying the fixed, pre-trained EfficientNet CNN to extract deep features is not specific to the lymph node dataset used in this study and contributes to generalizability. The approach can easily be applied to other image datasets.

It is also favorable that it does not require extensive computational resources compared to continued optimization of the CNN weights, such as in an “active” transfer-learning approach [40]. In contrast, task-specific feature extraction by updating the weights of a pre-trained CNN on the to-be-classified image dataset (active transfer-learning) has also shown promising results on different medical classification tasks [5,6,40].

### Study Limitations

Several limitations of this study merit consideration:

First, the investigated study population was retrospectively and randomly drawn from the clinical routine at a single-center comprehensive cancer center. It may reflect only a particular subpopulation of lung cancer patients. The generalization to different collections of lung cancer patients (i.e., other stages, other distributions of sub-entities) and other disease entities may be limited.

Second, as with all observational studies, the ability to conclude causality is limited, and results should be interpreted as hypothesis-generating.

Third, a suboptimal choice of the models’ respective hyperparameter configuration may have affected performance. Hence, this, and not solely model inputs, could have caused performance differences.

Fourth, the choice of the ground truth outcome (FDG-uptake) is generally accepted as the non-invasive reference standard for detecting lymph node metastases but may exclude rare or small lymph node metastases or inflammatory benign lymph nodes (e.g., in the case of tumor-associated pneumonia or chronic nicotine abuse) and does not equal the diagnostic performance of invasive methods [1,2,22,23]. On the other hand, transbronchial biopsy is also limited by too small tissue probes, and histology reports after resection provide the number of metastatic lymph nodes but not the exact anatomic location for coregistration with the imaging. However, limiting the study population to those with available histology from surgical lymph node dissection would have reduced the available study population to those that were a priori considered operable from the oncological point of view.

Concerning generalizability, we expect that combining traditional hand-crafted and deep features will also yield complementary information for other medical image classification tasks. However, before the deployment of a model in clinical routine, it would also require external validation, which is beyond the scope of this study.

## 5. Conclusions

In conclusion, both traditional radiomics features and transfer-learning deep radiomics features provide relevant and complementary information for classifying lymph nodes in lung cancer staging. Applying the radiomics approach to CECT could improve the nodal staging in lung cancer if FDG-PET/CT is unavailable, but metabolic FDG-PET/CT might even be the best non-invasive imaging technique.

## Figures and Tables

**Table 1:** Radiomics feature sets compared in this study.

(i)	Traditional hand-crafted shape, first-order, and higher-order features were extracted from the VOI in the respective CECT images using the AutoRadiomics application (https://github.com/pwoznicki/AutoRadiomics (accessed on 20 May 2023); [16]) as a wrapper for the pyradiomics package.
(ii)	A transfer-learning approach with a 2D-CNN to extract deep features (i.e., features considered relevant in an image classification task in a different domain) was applied. The output of the first k layers of an EfficientNet [17] pre-trained on the ImageNet database was aggregated and used as tabular deep features for machine learning classification. In detail, we masked the original image using the respective segmentation of a lymph node, cropped it to the bounding box of the lymph node segmentation, and finally rescaled it to match EfficientNet’s input dimensions. We rescaled the *z*-axis of the images to 10 pixels (the median *z*-axis length of all lymph nodes). We took the output of a forward pass through the first k convolutional layers of EfficientNet17 and applied an average pooling operation to get a number of features equal to the filters in the respective layer. The depth k of the final layer was considered a hyperparameter and optimized along with the other hyperparameters.
(iii)	Hybrid radiomics: a combination of transfer-learning CNN features from (ii) with traditional hand-crafted first-order and shape features from (i).

**Table 2:** Characteristics of the study population. Values are given as the mean (standard deviation) for continuous variables and as the count (relative percentage) for categorical variables.

	Total
	N = 100
Age (years)	65 (10)
Sex	
male	51 (51%)
female	49 (49%)
Smoker	
yes	64 (64%)
no	18 (18%)
N/A	18 (18%)
Therapy	
neoadjuvant chemotherapy	4 (4%)
adjuvant chemotherapy	16 (16%)
surgery	30 (30%)
definitive radiotherapy	71 (71%)
immunotherapy	4 (4%)
Side of primary tumor	
both sides	1 (1%)
right	47 (47%)
left	52 (52%)
Histology of primary tumor	
adenocarcinoma	60 (60%)
adeno-squamous carcinoma	1 (1%)
large cell neuroendocrine carcinoma	2 (2%)
unspecific non-small-cell lung cancer	3 (3%)
squamous cell carcinoma	21 (21%)
small-cell lung cancer	8 (8%)
unclear	5 (5%)
Metastasis at initial diagnosis	
yes	43 (43%)
no	53 (53%)
N/A	4 (4%)
Outcome (survival 07/2022)	
yes	22 (22%)
no	25 (25%)
N/A	51 (51%)
lymph node count per patient	27 (14)
percentage of round lymph nodes	3% (6%)
percentage of calcified lymph nodes	1% (3%)
percentage of inhomogeneous lymph nodes	2% (6%)
percentage of PET-positive lymph nodes	15% (25%)

Legend: N/A = information not available; PET = positron emission tomography.

**Table 3:** Classification results for “traditional” hand-crafted, deep, and hybrid features.

Metric	AUC	Brier Score (BS)	Scaled BS [%]		
Model					
Logit: (i) Radiomics data	0.857 (0.828–0.865)	0.112 (0.109–0.115)	30.8 (28.7–32.9)	0.76 (0.711–0.799)	0.803 (0.782–0.823)
Logit: (ii) Deep Radiomics data	0.788 (0.779–0.796)	0.137 (0.133–0.14)	15.7 (14.3–17)	0.784 (0.764–0.806)	0.72 (0.696–0.741)
Logit: (iii) Shape/First-order Features + Deep Radiomics	0.868 (0.861–0.875)	0.106 (0.102–0.109)	34.8 (33.2–36.4)	0.825 (0.789–0.861)	0.771 (0.735–0.807)
Random Forest: (i) Radiomics data	0.839 (0.831–0.847)	0.112 (0.109–0.116)	30.6 (28.7–32.4)	0.72 (0.698–0.744)	0.811 (0.788–0.831)
Random Forest: (ii) Deep Radiomics data	0.801 (0.793–0.809)	0.131 (0.128–0.135)	18.9 (17.5–20.2)	0.774 (0.755–0.792)	0.728 (0.71–0.745)
Random Forest: (iii) Shape/First-order Features + Deep Radiomics *	0.871 (0.865–0.878)	0.104 (0.101–0.107)	35.8 (34.2–37.2)	0.794 (0.764–0.824)	0.793 (0.764–0.823)

Legend: AUC = area under the receiver operating characteristic curve; BS = Brier score. * Overall, the best hyperparameter configuration was: random_trees = 890, min_sample_leaf = 5, criterion = ‘gini’, max_depth = None, min_sampl_decrease = 0.0, bootstrapes_split = 2, min_weight_fraction_leaf = 0.0, max_leaf_nodes = None, min_impurity = True, and CNN-depth = 3.

## Data Availability

The data presented in this study are available on reasonable request from the corresponding author. The data are not publicly available due to privacy restrictions according to German law.

## Supplementary Materials

The following supporting information can be downloaded at: https://www.mdpi.com/article/10.3390/cancers15102850/s1, Figure S1: Calibration and discrimination for classification of PET-positivity for different radiomics approaches. Results of xgboost are shown. In the lowess-smoothed calibration plot (first row), the observed outcome frequency is plotted against the predicted outcome probability. The closer the curve is to the diagonal, the better the calibration. The receiver-operating-characteristic (second row) plots the true positive rate against the false positive rate by varying thresholds (not shown). Discrimination is best for the curve that is closest to the left upper corner. Legend: PCA = principal component analysis; Table S1: Classification results using XGBoost for “traditional” hand-crafted, deep and hybrid features.

## Abbreviations

CECT	contrast-enhanced CT
AUC	Area under the receiver operating curve
ROC	receiver operating curve
BS	Brier score
SBS	Scaled Brier score
^18^FDG	^18^F-fluorodeoxyglucose
PET/CT	positron emission tomography/computed tomography
CNN	Convolutional neural network
