# Supplementary material for: Transfer-Learning Deep Radiomics and Hand-Crafted Radiomics for Classifying Lymph Nodes from Contrast-Enhanced Computed Tomography in Lung Cancer
Source: Cancers (Basel). 2023 May 21;15(10):2850. doi: 10.3390/cancers15102850 (PMC10216416; doi:10.3390/cancers15102850)
Supplement: Supplementary file 1 [file cancers-15-02850-s001.zip › cancers-2339462-supplementary.pdf]

Online Supplements

Transfer-Learning Deep Radiomics and Hand-Crafted Radiomics for Classifying Lymph Nodes from Contrast-Enhanced Computed Tomography in Lung Cancer

Fabian Christopher Laqua [1,*], Piotr Woznicki [1], Thorsten A. Bley [1], Mirjam Schöneck [2], Miriam Rinneburger [2], Mathilda Weisthoff [2], Matthias Schmidt [3], Thorsten Persigehl [2], Andra-Iza Iuga [2,†] and Bettina Baeßler [1,†]

[1] Department of Diagnostic and Interventional Radiology, University Hospital Würzburg, University of Würzburg, 97080 Würzburg, Germany; woznicki_p@ukw.de (P.W.); bley_t@ukw.de (T.A.B.); baessler_b@ukw.de (B.B.)
[2] Institute of Diagnostic and Interventional Radiology, Medical Faculty and University Hospital Cologne, University of Cologne, 50937 Cologne, Germany; mirjam.schoeneck@uk-koeln.de (M.S.); miriam.rinneburger@uk-koeln.de (M.R.); mathilda.weisthoff@uk-koeln.de (M.W.); thorsten.persigehl@uk-koeln.de (T.P.); andra.iuga@uk-koeln.de (A.-I.I.)
[3] Department of Nuclear Medicine, Medical Faculty and University Hospital Cologne, University of Cologne, 50937 Cologne, Germany; matthias.schmidt@uk-koeln.de
* Correspondence: laqua_f@ukw.de; Tel.: +49-9301-201-34288; Fax: +49-9301-201-634001
† These authors contributed equally to this work.

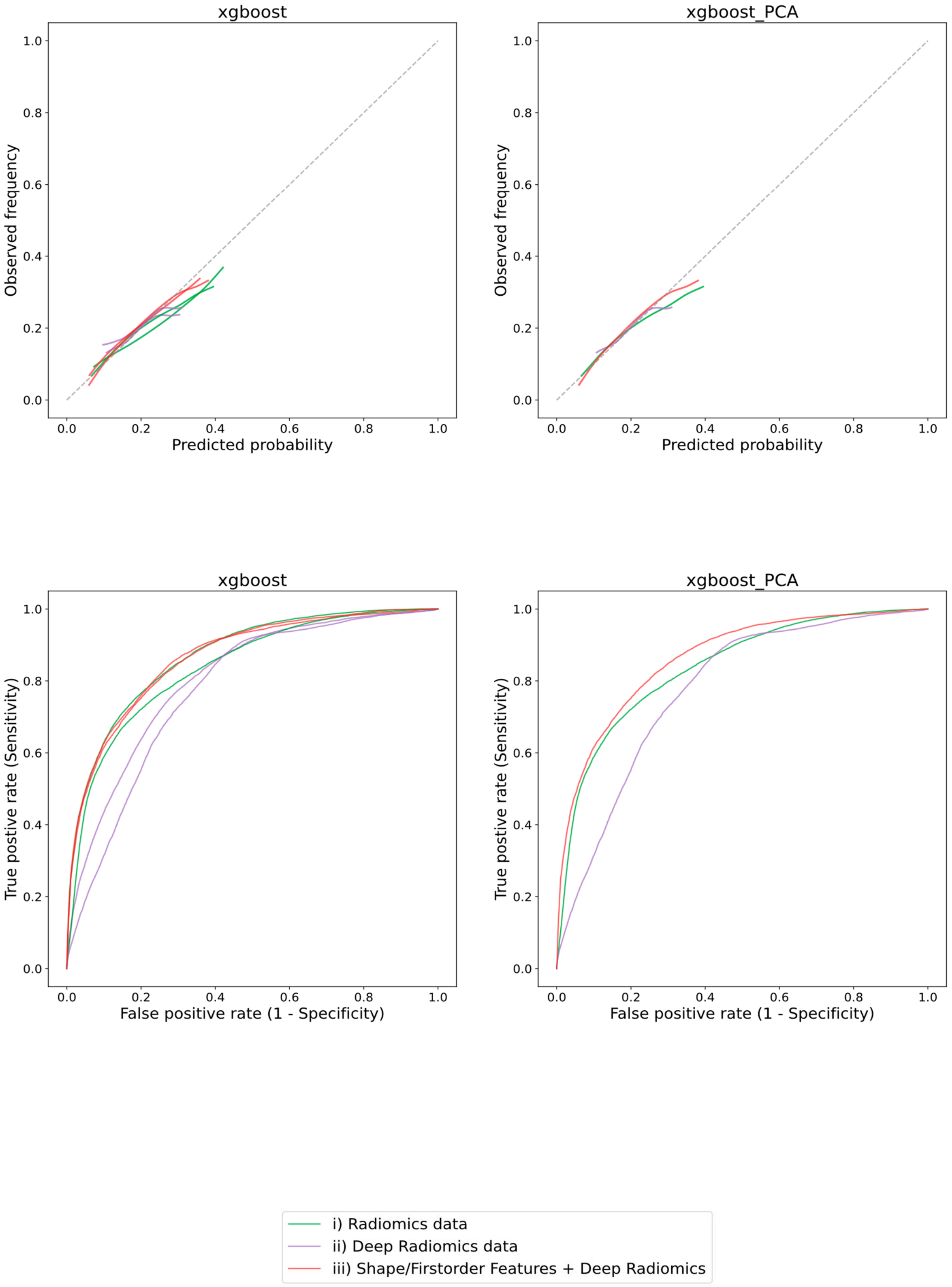

Figure S1. Calibration and discrimination for classification of PET-positivity for different radiomics approaches. Results of xgboost are shown. In the lowess-smoothed calibration plot (first row), the observed outcome frequency is plotted against the predicted outcome probability. The closer the curve is to the diagonal, the better the calibration. *The receiver-operating-characteristic (second row) plots the true positive rate against the false positive rate by varying thresholds (not shown). Discrimination is best for the curve that is closest to the left upper corner. Legend: PCA = principal component analysis.*

Table S1. Classification results using XGBoost for "traditional" hand-crafted, deep and hybrid features.

Metric<br>Feature type<br>Model	AUC<br>Radiomics	Brier score (BS)<br>Radiomics	R^2 (scaled BS)<br>Radiomics	Sensitivity<br>Radiomics	Specificity<br>Radiomics
**XGBoost: i) Radiomics data**	0.86 (0.838-0.875)	0.119 (0.116-0.121)	26.6 (25.1-28.3)	0.76 (0.711-0.799)	0.803 (0.782-0.823)
**XGBoost: ii) Deep Radiomics data**	0.807 (0.801-0.812)	0.129 (0.126-0.131)	20.5 (19.5-21.6)	0.784 (0.764-0.804)	0.705 (0.684-0.725)
**XGBoost: iii) Shape/Firstorder Features + Deep Radiomics**	0.868 (0.862-0.873)	0.105 (0.102-0.108)	35 (33.7-36.5)	0.821 (0.794-0.845)	0.763 (0.739-0.789)
**XGBoost + PCA: i) Radiomics data**	0.839 (0.832-0.845)	0.121 (0.118-0.125)	25 (23.2-26.7)	0.723 (0.703-0.747)	0.817 (0.795-0.836)
**XGBoost + PCA: ii) Deep Radiomics data**	0.776 (0.77-0.782)	0.138 (0.136-0.141)	14.6 (13.5-15.6)	0.853 (0.828-0.876)	0.609 (0.585-0.636)
**XGBoost + PCA: iii) Shape/Firstorder Features + Deep Radiomics**	0.864 (0.857-0.87)	0.107 (0.104-0.11)	34 (32.6-35.5)	0.798 (0.769-0.825)	0.775 (0.747-0.801)
